# Reconsideration at Field Scale of the Relationship between Hydraulic Conductivity and Porosity: The Case of a Sandy Aquifer in South Italy

**DOI:** 10.1155/2014/537387

**Published:** 2014-08-07

**Authors:** Carmine Fallico

**Affiliations:** Department of Civil Engineering, University of Calabria, 87036 Rende, Italy

## Abstract

To describe flow or transport phenomena in porous media, relations between aquifer hydraulic conductivity and effective porosity can prove useful, avoiding the need to perform expensive and time consuming measurements. The practical applications generally require the determination of this parameter at field scale, while most of the empirical and semiempirical formulas, based on grain size analysis and allowing determination of the hydraulic conductivity from the porosity, are related to the laboratory scale and thus are not representative of the aquifer volumes to which one refers. Therefore, following the grain size distribution methodology, a new experimental relation between hydraulic conductivity and effective porosity, representative of aquifer volumes at field scale, is given for a confined aquifer. The experimental values used to determine this law were obtained for both parameters using only field measurements methods. The experimental results found, also if in the strict sense valid only for the investigated aquifer, can give useful suggestions for other alluvial aquifers with analogous characteristics of grain-size distribution. Limited to the investigated range, a useful comparison with the best known empirical formulas based on grain size analysis was carried out. The experimental data allowed also investigation of the existence of a scaling behaviour for both parameters considered.

## 1. Introduction

Porosity is the fraction of the total volume of rock that is not occupied by the solid constituents [1]; therefore this parameter more than any other one characterizes the medium, allowing the two components (solid and void) that constitute it to be estimated. These components usually have variable ratios for the different porous media and also for the same typology. With variable size of solid particles they originate a complex hierarchy, characterized by physical and geometrical properties still the subject of study and open to different interpretations. The flow and transport phenomena depend strongly on this parameter, both for pore dimension and the connectivity and continuity of the network that they create. These circumstances suggest considering not the total but the effective porosity, which is the fraction of pores that can contribute to fluid flow, considering only the connected pores [1–7].

A relation between the flow or transport parameters and effective porosity characterizing the medium structure is very important, because it means avoiding often expensive and time consuming analysis and measurements. Some researchers investigated relations of this type between electrical conductivity and porosity [8–12], velocity of sound or seismic waves and porosity [13–16], and hydraulic conductivity and grain size distribution [17–22] in porous media. To determine the hydraulic conductivity of an aquifer empirical and semiempirical formulas that relate this parameter to the effective porosity are often used. In this way, measuring the hydraulic conductivity is enough to determine the effective porosity value on soil samples extracted from the aquifer in the laboratory and, finally, using the formula considered more suitable to the specific case, to determine the corresponding hydraulic conductivity value. The parameter values measured in the laboratory can often prove scarcely reliable for the disturbance that occurs during the soil drilling and sampling operations [22, 23]. With reference to direct measurements of hydraulic conductivity carried out in laboratory by permeameter, the problem of the scarce reliability of the values obtained is greater for sandy than clayey and silty soils. In fact, for more cohesive soils the samples are minimally disturbed and so the values measured show commonly good reliability [21, 24]. Hydraulic conductivity values obtained by field measurements depend also on the aquifer characteristics. In fact, some authors [25], performing field measurements on cementified and consolidated soils, found hydraulic conductivity values lower than those obtained for soils without these characteristics. Moreover, some authors [22] showed that laboratory measurements on small volumes of soil samples can provide underestimated hydraulic conductivity values, compared to those obtained by field measurements. The difference between the hydraulic conductivity values measured in laboratory and those measured in field is also justified, because the laboratory methods commonly determine the vertical hydraulic conductivity, while the field methods determine the horizontal hydraulic conductivity which is generally greater than that of vertical hydraulic conductivity [22, 26]. Further concerns arise because the values of the parameters obtained for each soil sample in the laboratory cannot be extended to larger aquifer volumes, nor to the whole aquifer, but their validity remains confined to the measurement volume, namely, that of the sample. Mainly it should be noted that the measurements are in any case affected by the heterogeneity of the sample and this manifests itself in different ways at smaller rather than higher scales. At the laboratory scale the influence of the soil heterogeneity is mainly related to the pore sizes and their shape, that is, the presence of macropores, whereas at larger scales (in the field) it is essentially related to the connectivity and tortuosity of pores in which the water flow occurs within the porous medium [27–31].

Moreover it is of fundamental importance to clarify whether the hydraulic conductivity value to be determined must be representative of a very limited aquifer volume or relative to a more or less wide portion of this. Considering very limited volumes, retaining reservations relating to the aspects mentioned above, the measurements performed on soil samples in the laboratory can also be taken into account. For larger aquifer volumes the laboratory measurements cannot be considered representative, because they were performed at a scale different from that of interest. In the latter case of larger scales, the parameters in question must necessarily be determined by field methods, which give representative values of the actual volume of the aquifer affected by the measurement and that require a determination of the indirect type of the parameters under consideration. These observations must be taken into consideration to determine the relationship between hydraulic conductivity (*k*) and effective porosity (*n*_*e*_). Relationships of this type are determined experimentally on the basis of *k* and *n*_*e*_ measurements carried out for assigned soil types. If these measures are carried out in the laboratory, the relationship between *k* and *n*_*e*_ presents representativeness limited to the laboratory scale, whereas to obtain a relationship representative of the larger aquifer volumes this relation should be determined on the basis of field measurements. Therefore, the knowledge of the purposes and consequently of the scale of interest to which the hydraulic conductivity of an aquifer must be referred is of primary importance, since it influences the choice of the particular relationship between *k* and *n*_*e*_ to be used. The choice of the field measurement method is also of great importance because it determines the measurement scale. Among the conventional field measurement methods the slug tests are relative to small scales, while pumping tests, recovery tests, and tracer tests result in a reference to larger scales.

The aim of the present work is to investigate a relation between *k* and *n*_*e*_, showing the importance of defining at field scale this functional dependence in a specific and reliable manner. For this purpose experimental values of both the parameters *k* and *n*_*e*_ were obtained by field measurements. In this way a new experimental relation was here determined, showing greater representativeness than those obtained using empirical relations that do not take into account the portion of the aquifer to which *k* and *n*_*e*_ must be referred. The relationship obtained in this way is valid not only for the porous medium investigated but also for porous aquifers with similar characteristics. Therefore in the present study the influence of the effective porosity on the hydraulic conductivity to field scale is investigated, considering the confined aquifer of the Montalto Uffugo (Italy) test field, where several measurements of the flow and structural parameters *k* and *n*_*e*_ were carried out, using different field measurement methods and involving increasing volumes. Moreover, the scaling effect for the considered parameters was investigated on the basis of the experimental data. This allows verification of the scaling behavior of the hydraulic conductivity, which was already investigated in field and numerical studies [32–35], and mainly of the porosity, about which less is known [36–39].

## 2. Experimental Site

**Figure 1:**
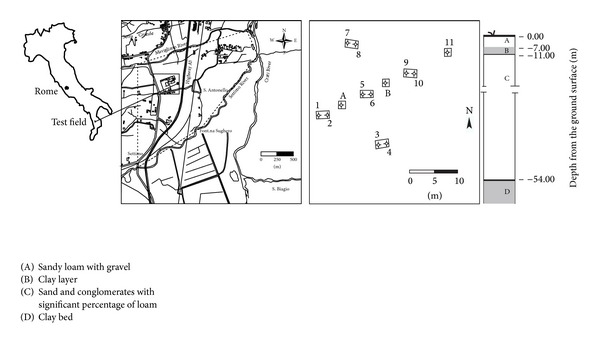
Planimetrical schematization of the test field. Even numbers show the wells relative to the shallow aquifer, odd numbers show the wells and A and B, the piezometers relative to the confined aquifer.

The investigation was carried out on the confined aquifer of the Montalto Uffugo (Italy) test field. The area under consideration has the geological characteristics of a recently formed valley, with slightly consolidated conglomeratic and sandy alluvial deposits of the Calabrian epoch. This formation is of relatively limited thickness. The stratigraphic scheme of the test field shows the interposition of a clay layer, with about 4 m of thickness, between a covering layer of alluvial deposits and a consistent sand bank, with variable and significant percentages of silt in the various levels and traces of clay in the part nearest to the bottom, which reaches a depth of as far as 55 m below the ground surface, where there is the substratum of the aquifer constituted of a clay bank of very large thickness [40]. The presence of the clay layer between the overlying alluvial layer and the underlying sand bank causes the formation of two aquifers: one superficial, unconfined, and another, deep, of the confined type. The test field has eleven wells and two piezometers. Five of the wells, marked with even numbers and 8 m deep, affect only the shallow aquifer. The other six wells, marked with odd numbers, affect the confined aquifer, below the clay layer; of the latter, five are 40 m deep and only one (well number 11) is 57 m deep and is completely penetrating, going for about 2 m into the bottom clay. Two piezometers A and B are both entirely penetrating. Piezometer A is 5 m from both well number 1 and well number 5; analogously piezometer B is 5 m from both well number 5 and well number 9. Indications about the stratigraphy of the test field area and the planimetrical layout of the wells and piezometers are shown in the scheme of Figure 1. In Table 1, for each well and piezometer relative to the confined aquifer of the test field, the corresponding identification number, the altitude of the well-top above sea level, the depth from the ground level, and the screen length are summarized.

## 3. Methodology

To obtain a relationship between *k* and *n*_*e*_ valid for a scale greater than that of the laboratory, specifically for the field scale, it is necessary to perform initially a careful laboratory characterization of the porous medium under consideration. In any case, this requires the availability of a number of soil samples, the performance on each of these of the particle size analysis, and, even if not strictly required, careful laboratory measurements of the total and effective porosity and the hydraulic conductivity. Thereafter it is necessary to carry out a series of field measurements to determine the greatest possible number of hydraulic conductivity and effective porosity values. The field measurement methods to consider may be the ones most commonly used, like slug tests, pumping tests, and tracer tests, each of which takes into account different aquifer volumes and field scales (small, medium, and large). These methods give an indirect measurement, because they are able to appraise the examined parameter utilizing relations with other easily measurable parameters. The field measurements, also if involving aquifer systems with scarcely known aspects and uncertain initial and boundary conditions, prove to be very representative of the examined porous media. In fact they refer commonly to large measurement volumes, on which the influence of the heterogeneity is not much evident, and in an averaged manner, meaning that, with reference to unconsolidated sand formations, single heterogeneities, such as macropores or fissures, are irrelevant. Therefore a large range of aquifer measurement volumes to field scale was considered for the parameter measurements examined in this study and an experimental relation between *k* and *n*_*e*_ was determined. Of course, the validity of a relation as

(1) $$\begin{array}{c} k=k\left(n_{e}\right) \end{array}$$

is limited to the aquifer considered and those with similar soil type, highlighted by the particle size analysis. Moreover in the present paper the same relation was also investigated utilizing the grain size distribution theory. For this purpose the general model of Vuković and Soro [41] was assumed, represented by the following equation:

(2) $$\begin{array}{c} k=\frac{g}{\nu }Cf\left(n\right)d_{e}^{2}, \end{array}$$

where *k* is the hydraulic conductivity of saturated porous media [LT^−1^], *g* the acceleration of gravity [LT^−2^], *ν* the kinematic viscosity [L^2^T^−1^], *C* a general coefficient [-], *n* the total porosity [-], *f*(*n*) the porosity function which defines the relationship between the real and modeled porous media or the degree of material compactness, and *d*_*e*_ the effective grain diameter [L]. This general model may be found in numerous commonly used empirical and semiempirical formulae, showing different governing factors for *k*. Finally, with the data sets related to field measurements of the parameters in question available, it was convenient to verify the existence of a scaling law, for both *k* and *n*_*e*_. In the present study this was done assuming the model proposed by Schulze-Makuch and Cherkauer [42] to describe the scale dependence of aquifer parameters for various geological units, which is based on a power-type relationship and is expressed, with reference to the hydraulic conductivity, as

(3) $$\begin{array}{c} k=cs^{m}, \end{array}$$

where *k* is hydraulic conductivity [LT^−1^]; *s* the scale parameter (volume [L^3^] or its characteristic dimension [L]), *c* parameter related to the heterogeneity of medium with the same dimensions as *k*, and *m* scaling index, which take into account the fluid-flow type in porous media and the effective dimensions of the measurement scale. A similar relationship can be considered also for the porosity.

### 3.1. Measurements at the Laboratory Scale

**Figure 2:**
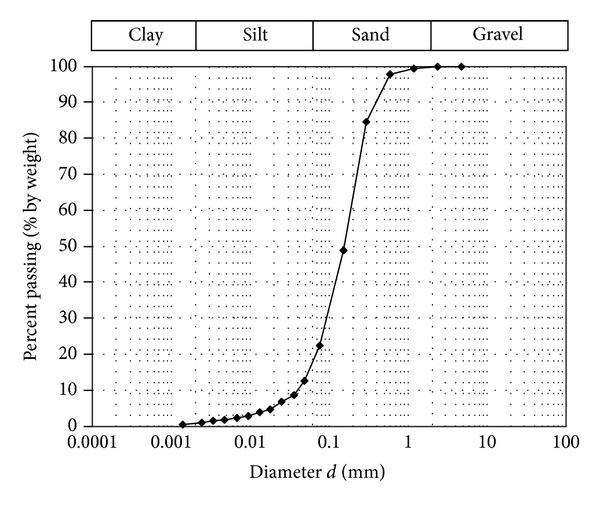
Typical grain size distribution curve (sample number 14 with midpoint depth equal to 47.45 m on the drilling column of the piezometer A).

To characterize the considered soil aquifer a careful grain size analysis was carried out in laboratory on thirty-two undisturbed soil samples, extracted at different depths, between 11 m and 55 m from the ground surface, from the drilling columns of two piezometers A (n. 18 samples) and B (n. 14 samples), from which the meaningful parameter values of soil identification were obtained. For each sample the effective grain diameters *d*_10_ and *d*_60_ (resp., the particle size for which 10% and 60% of the sample are finer than) [L] and the coefficient of grain uniformity (*U* = *d*_60_/*d*_10_) [-] were determined. The values of these parameters, with the percentage of clay, silt, and sand, are shown in Table 2. The grain size analysis shows that samples are composed mainly of sand. Often silt is a considerable portion of samples. The amount of clay in most of the samples was found to be negligible and only for some of these it was significant. As an example, a typical grain size distribution curve, relative to the sample number 14, with midpoint depth from the ground surface on the drilling column of the piezometer A equal to 47.45 m, is shown in Figure 2. For each of the 32 soil samples previously considered both the total (*n*) and effective (*n*_*e*_) porosity were measured. The total one (*n*) was measured utilizing a laboratory method [43, 44] by the following relation:

(4) $$\begin{array}{c} n=1-\frac{\rho_{\text{bulk}}}{\rho_{\text{grain}}}, \end{array}$$

where *ρ*_bulk_ is the bulk mass density [ML^−3^] and *ρ*_grain_ the particle mass density [ML^−3^].

**Figure 3:**
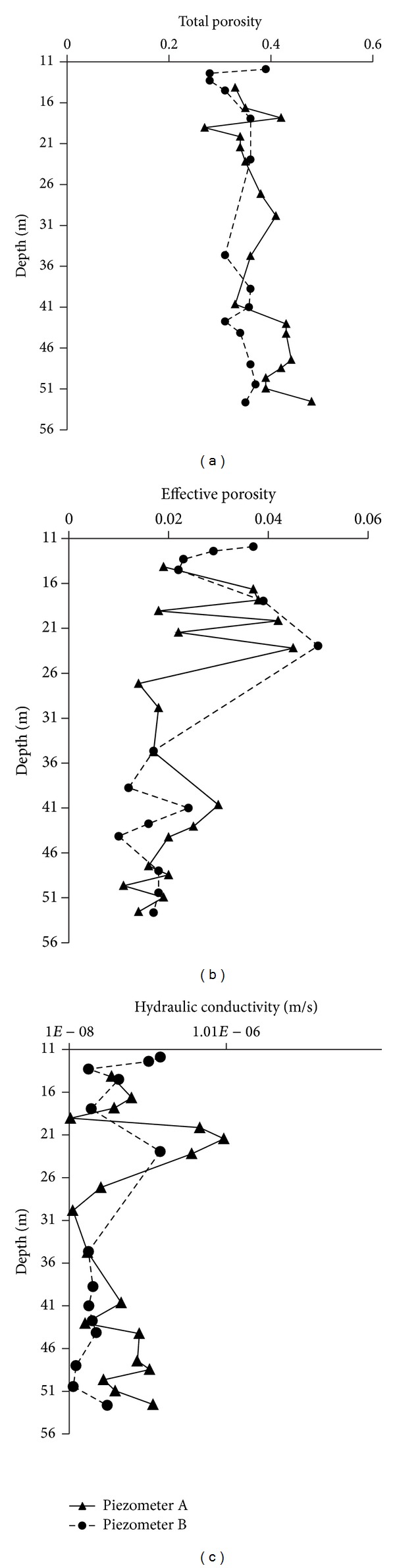
Variation of total porosity (graph a), effective porosity (graph b), and hydraulic conductivity (graph c) with depth, along the drilling columns of piezometers A and B.

The effective porosity (*n*_*e*_), considered as saturated water content minus residual water content,

(5) $$\begin{array}{c} n_{e}=n-\frac{V_{w}}{V}, \end{array}$$

where *V* is the total volume [L^3^] and *V*_*w*_ the water volume which cannot be drained by gravity [L^3^] [45], was measured under equilibrium conditions at 33 kPa of suction [2, 3]. Moreover for each of thirty-two undisturbed soil samples examined the respective hydraulic conductivity was measured by flow cells, used as constant head permeameter [46]. The variability of *n*, *n*_*e*_, and *k* was investigated along the entire thickness of the aquifer for both drilling columns of the two piezometers A and B. The vertical profiles (a), (b), and (c) of Figure 3 show that the examined aquifer can be considered without significant stratifications. In fact the vertical variations of *n*, *n*_*e*_ and *k* result contained in a fairly limited range for both the considered drilling columns.

### 3.2. Measurements at the Field Scale

#### 3.2.1. Hydraulic Conductivity Measurements

The knowledge of the hydraulic conductivity and its modality of variation in a porous aquifer is often very important for hydrologists to determine and model the flow and the transport processes. Having a precise knowledge of the geometry and hydrogeologic boundaries of the aquifer and wishing only to refer to measurement volumes of aquifer at the field scale, that is much greater than those of the laboratory samples, only hydraulic conductivity values measured in the field by slug tests, aquifer tests and tracer tests were here considered.

Since absence of stratification may be found in the aquifer here examined, conventional slug tests were performed, without recourse to particular technologies, as packer systems [47, 48] or direct push (DP) multilevel slug tests [49–52] particularly suitable for stratified aquifers [22]. The slug tests were carried out following the guidelines suggested by Butler et al. [53] and Butler [54]. More than three measurements with different initial head were performed for each considered well; each measurement was repeated with the same head for three times; the slug was introduced in a near-instantaneous fashion; the data were acquired by automatic devices; the data analysis method was chosen suitably for site conditions and particular attention has been paid to performing analysis. There were altogether fifteen *k* values measured by slug tests. These tests were carried out on the entirely penetrating well number 11 and on the completely penetrating piezometers A and B also. All these wells and piezometers are relative to the considered confined aquifer. The water volumes *V* rapidly admitted in the columns during the tests are included between 0.003 m^3^ and 0.030 m^3^ for the piezometers A and B and between 0.005 m^3^ and 0.040 m^3^ for well number 11. The resulting water level variations were measured by proper pressure transducers at fixed times, beginning from that of maximum water elevation level till the restoration of the undisturbed level [54]. For each test the geometry of the aquifer-well system was considered well known. In fact, among the initial and boundary conditions that determine the choice of the test interpretation method, it is very important to note that for the examined tests the undisturbed piezometric level was always placed above the well-screened zone and then not intersecting this [54]. The drawdown-time data sets obtained in this way for the entirely penetrating well number 11 and the piezometers A and B were analyzed by the Cooper method [55]. This method allowed determination of the radial component of hydraulic conductivity and the specific storage (*S*_*S*_). The aquifer volume involved in the measurement for these tests was obtained determining the corresponding radius of influence in two ways, by the Barker and Black [56] method, which is an extension of the Cooper method, and assuming a value equal to 200 times the effective radius of well screen [57]. The slug tests here considered were multiwell, namely, the water level measurements were carried out at the same time also in all the wells and piezometers of the test field relative to the confined aquifer, which are aligned with reference to the prevailing direction of water flow, for a maximum distance equal to 29 m [40]. In this way for each slug test it was possible to perform verification of the radius of influence values obtained by the two mentioned methods and to take as more representative the values obtained by the method suggested from U.S. Department of Navy [57], which resulted very close to those obtained on the basis of the field measurements.

In the present study the results of fifteen pumping tests, carried out on the confined aquifer of the Montalto Uffugo test field over several years, were considered. All the tests were performed to constant pumping rate between 5.7*·*10^−4^ m^3^/s and 4.55*·*10^−3^ m^3^/s and for time ranges between 23 and 94.8 hours. During the tests the data, measured simultaneously at the different wells and piezometers with well-known distances from the pumping well, were automatically acquired by proper devices able to measure and memorize the values of the water level by pressure transducers, time, and temperature at fixed time ranges.

**Figure 4:**
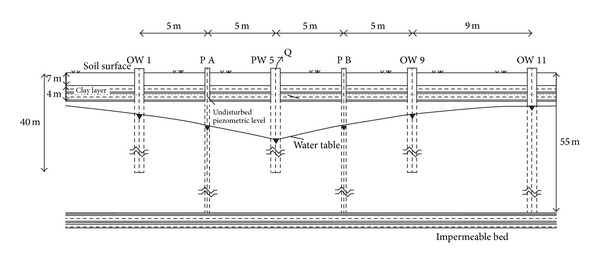
Behavior variation of the aquifer considered from confined to unconfined during the pumping.

For the fifteen pumping tests, carried out in transient state conditions, the drawdown-time data were analyzed by the Neuman [58] and Jacob [59] methods, considering the initial and boundary conditions and the geometry of the system well known and taking into account that during the pumping the aquifer behaviour passed from confined to unconfined, as shown in Figure 4, because in the absence of pumping and in the beginning of this the aquifer is under very limited hydraulic head. The values of the radius of influence (*R*) were determined for the pumping tests by the semiempirical formula of Kusakin [60]:

(6) $$\begin{array}{c} R=1.9{\left(\frac{B\cdot k\cdot t}{S_{y}}\right)}^{1/2}, \end{array}$$

where *R* is the radius of influence [L], *B* the aquifer thickness [L], *S*_*y*_ the specific yield [-], *k* the hydraulic conductivity [LT^−1^], and *t* the time corresponding to the pumping length [T].

In order to obtain as much data as possible, five tracer tests were also considered, carried out on the confined aquifer of the Montalto Uffugo test field in the period between 1996 and 1998. These tracer tests were performed during a pumping test, using number 1 as the tracer inflow well and number 5 as the pumping and observation well. These two wells are 10 m apart. For all the tests NaCl was used as the tracer in well number 1 in a solution volume of 0.4 m^3^, with an NaCl concentration of 200 kg/m^3^. The tracer inflow was performed in a short time for each test. Moreover, in tracer inflow well number 1 the introduced solution was suitably homogenized along the well column by a recirculation circuit, withdrawing water from the bottom of the well by a pump and entering it in the upper part of the water column. The pumping rates, kept constant during each considered tracer test, are included in a range of 9*·*10^−4^ m^3^/s and 3.3*·*10^−3^ m^3^/s, while the respective durations ranged between 5.4 days and 34.84 days.

**Figure 5:**
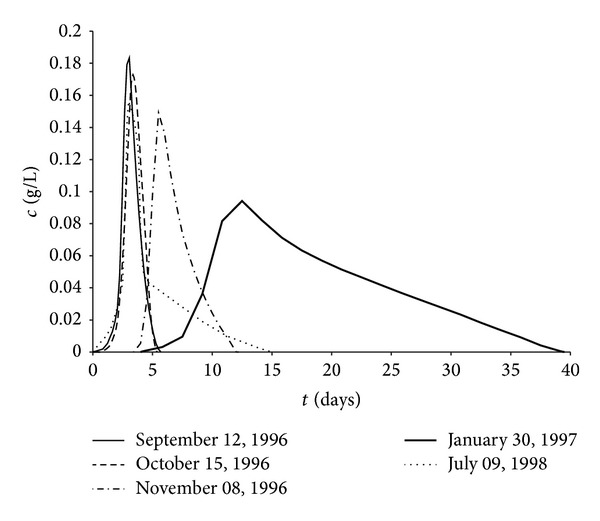
Experimental breakthrough curves for the considered tracer tests.

The sampling performed in well number 5 allowed determination of the breakthrough curves, shown in Figure 5, for the considered five tracer tests. During the pumping performed for each tracer test, flow in the aquifer was at steady-station conditions for drawdown-times data, which was analysed by the Dupuit [61] method, determining the transmissivity and therefore the hydraulic conductivity.

#### 3.2.2. Effective Porosity Measurements

The effective porosity (*n*_*e*_) values here determined were obtained indirectly by determining other parameters relative to specific flow conditions caused in the aquifer by the tracer tests, slug tests, and pumping tests carried out in the field and which also allowed the determination of the *k* values.

The fifteen slug tests considered also allowed the effective porosity to be determined. In fact, the Cooper method [55], applied to the considered confined aquifer, allowed the specific storage (*S*_*S*_) [L^−1^] to be determined [54]. Once this parameter is determined and its definition is recalled, it was possible to determine *n*_*e*_ by the following relation:

(7) $$\begin{array}{c} n_{e}=\frac{S_{S}-\gamma \cdot \beta_{S}}{\gamma \cdot \beta_{w}}, \end{array}$$

where *β*_*w*_ is the water compressibility [M^−1^LT^2^], *β*_*S*_ the solid phase compressibility [M^−1^LT^2^], and *γ* the water specific weight [M L^−2^T^−2^] and the other symbols were already specified. Regarding the slug tests, the variance of *S*_*s*_, equal to 1,74*·*10^−11^, and the relative standard error, equal to 1,20*·*10^−6^, can be assumed to represent the uncertainty of the specific storage. The impact of the uncertainty of *n*_*e*_ associated with *S*_*s*_ was assessed. For a variation of *S*_*s*_ between the minimum and maximum value of the corresponding data set, the variation of *n*_*e*_ around the average can be characterized by the variance and the standard deviation values equal to 1.92*·*10^−6^ and 1.39*·*10^−3^, respectively. In each case all the *n*_*e*_ values remain contained within a fairly narrow range, retaining the same order of magnitude.

The data analysis of the fifteen pumping tests, carried out with constant rate and transient state conditions previously discussed, allowed the specific yield (*S*_*y*_) [-] to be determined, still using the Neuman method. Therefore, considering that in unconfined aquifers the effective porosity can be assumed to be almost equal to the specific yield, since the elastic storage component gives a negligible contribution, it was possible also to obtain the effective porosity values for the considered pumping tests.

For the five tracer tests carried out on the confined aquifer of the Montalto Uffugo test field, once the hydraulic conductivity *k* is obtained by the formula of Dupuit, the Darcian velocity (*V*_*D*_) [LT^−1^] was also determined. The breakthrough curve analysis of the considered tracer tests allowed the correspondent effective velocity (*V*) [LT^−1^] in the aquifer to be determined [62]. Assuming the hypothesis of radial convergent flow, it was possible also to determine for each considered tracer test, in addition to the hydrodispersive parameters (Péclet number, dispersivity, and dispersion coefficient), the effective porosity by the following relation:

(8) $$\begin{array}{c} n_{e}=\frac{V_{D}}{V_{e}}=\frac{Q}{\pi rh}\cdot \frac{1}{V_{e}}=\frac{Q}{\pi r^{2}h}\cdot \frac{\int_{0}^{\infty }tC\left(t\right)dt}{\int_{0}^{\infty }C\left(t\right)dt}, \end{array}$$

where *Q* is the pumping rate, *h* the aquifer thickness, *r* the distance between the injection and the pumping wells, *t* the time, and *C*(*t*) the concentration value at *t* time [63, 64].

## 4. Results and Discussion

To characterize suitably the *k* and *n*_*e*_ data sets obtained by the different field measurement methods examined, a careful statistical analysis was performed, determining the meaningful parameters for each of them, as well as the minimum (min), maximum (max), mean, and median values. In addition, the variance (VAR), standard deviation (SD), standard error (SE), and variation coefficient (VC) were also estimated. The data number (*N*) for each obtained data set and the values of these parameters are shown in Table 3. The values of Table 3 show that, for the hydraulic conductivity *k*, the variance, standard deviation, standard error, and variation coefficient assume the largest values for set of tracer test results. The same occurs for the values of these statistical parameters relative to the effective porosity.

**Figure 6:**
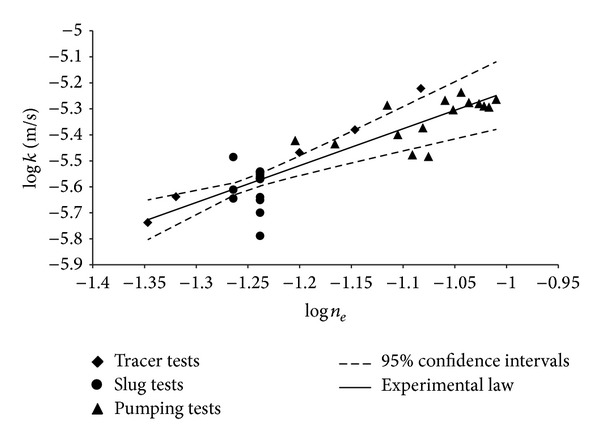
Experimental values of *k* and *n*_*e*_ for the different considered field measurement methods and relative best fitting curves representing the *k* = *k*(*n*_*e*_) law.

**Figure 7:**
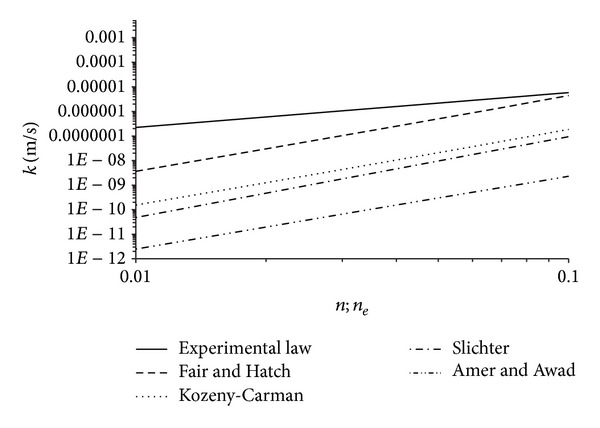
Trends of the experimental law *k* = *k*(*n*_*e*_) and semiempirical formulae of Kozeny-Carman, Slichter, Fair and Hatch, and Amer and Awad for the considered aquifer.

**Figure 8:**
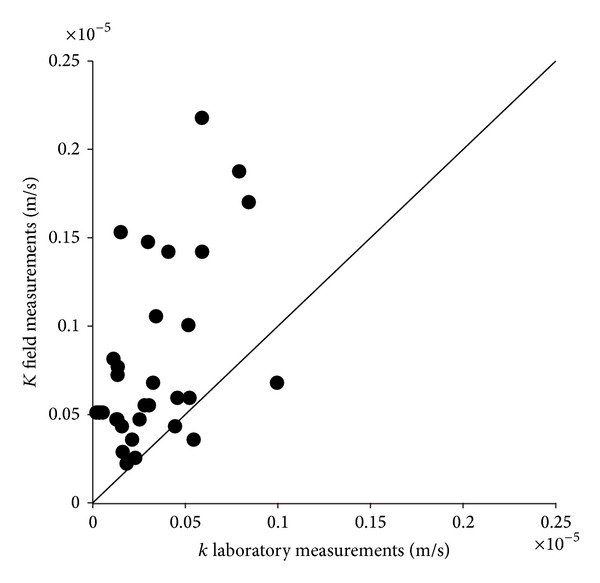
Comparison between hydraulic conductivity values measured for each soil sample in laboratory and in field for the correspondent *n*_*e*_ values.

**Figure 9:**
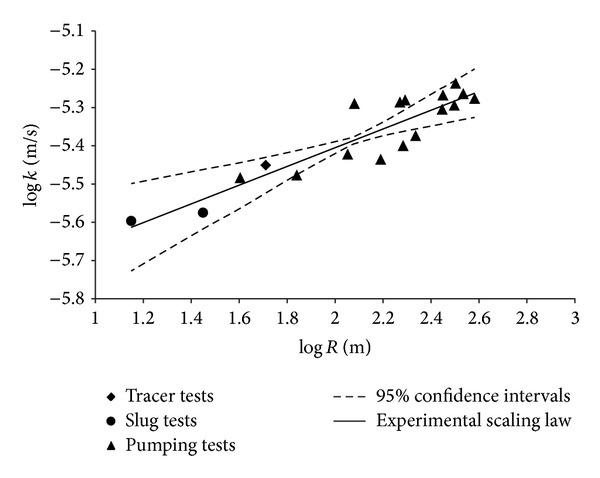
Trend of hydraulic conductivity versus scale for all field data sets.

The hydraulic conductivity and effective porosity values, measured in the field by the different methods above mentioned, allow an experimental relation to be found between the two considered parameters. The pairs of *k* and *n*_*e*_ values obtained by each field measurement method examined were considered, drawing on the suitable graph the corresponding points. Therefore it was possible to find the best fitting curve and the relation that describes it. This equation, giving the variation law of *k* versus *n*_*e*_ for the examined aquifer, with *k* expressed in m/s, is the following:

(9) $$\begin{array}{c} k=1.52\cdot 10^{-4}n_{e}^{1.418} \end{array}$$

showing a determination coefficient (*R* ^2^) equal to 0.780. In Figure 6, both axes are in logarithmic scale, and the *k* and *n*_*e*_ experimental values, obtained in the field by the different measurement methods considered, are shown. Moreover, in Figure 6 the best fitting curve relative to all the field data, described by (9), with corresponding 95% confidence intervals [65], is shown. The *R* ^2^ value, relative to this best fitting curve, states that (9) gives a good description of the *k* versus *n*_*e*_ trend. It is appropriate to point out that, since the characteristics of the system (aquifer-piezometer) are the same for both the piezometers A and B, all slug tests performed on these two piezometers gave, by (7), always the same *n*_*e*_ value. The same thing happened for the *n*_*e*_ values determined by slug tests on well number 11. Taking into account that the grain size analysis is certainly a good simplified method to characterize the soil hydraulic properties, rapid, and less expensive than field measurement methods [21], it is reasonable to propose experimental law (9) following the model proposed by Vuković and Soro [41], with explicit reference to the soil examined, characterized by the results of the grain size analysis shown in Table 2, or soils with similar characteristics. Therefore (9) can be also expressed in the following form:

(10) $$\begin{array}{c} k=\frac{g}{\nu }1.76\cdot 10^{-2}n_{e}^{1.418}d_{10}^{2}, \end{array}$$

where *d*_10_ is the particle size for which 10% of the sample are finer than [*L*] and the meaning of other symbols was already specified. Equation (10) allows the variability of the *k* = *k*(*n*_*e*_) law to be determined varying the values of *d*_10_ in the range investigated by grain size analysis. The trend, described from the considered aquifer by (9) or (10), was compared, relatively to the investigated range, with some of the more commonly used empirical and semiempirical formulae based on the grain size analysis, as, for example, those of Kozeny [66] and Carman [67, 68], Slichter [69], Amer and Awad [70], and Fair and Hatch [71] which give *k* values as a function of the porosity. The log-log graph of Figure 7 shows this comparison and highlights that the empirical and semiempirical formulas taken into consideration provide, for the investigated range, values of *k* smaller than those given by the relation here determined experimentally, represented by (9) or (10). This can be assumed as a consequence of the fact that the aforementioned empirical equations take into account the total and not the effective porosity, as instead the relationships (9) and (10), making them not suitable for a proper estimate of *k*. Moreover, they were determined by laboratory measurements, while relation (9) or (10) was determined by field measurements. In fact, the remarks quoted previously are valid for laboratory measurements and should be taken into account. Specifically, the possible alteration of the soil structure, the packing and the compaction degree of the grains during the sampling [22], validity limited to laboratory scale, and, moreover, the influence of the vertical heterogeneity on the parameters considered, specifically for the hydraulic conductivity, as previously highlighted. This last condition is also highlighted by Figure 8, showing a comparison between the *k* values determined by (9) or (10) with those measured directly in laboratory on the soil samples and confirming that the latter are less than the former [21, 23]. Therefore, considering only field measurements, the significance of the heterogeneity, and then of the scale, influence on the parameters examined suggested the scaling behaviour of these should be investigated, assuming as a representative scale parameter (*s*) both the radius of influence (*R*) and the cylindrical aquifer volume (*V*) involved in the measurement, with radius *R* and height equal to the thickness of the aquifer. For each measurement method here considered the correspondent values of the scale parameter (*s*), expressed in terms of both radius of influence (*R*) and volume (*V*), are shown in Table 4. Regarding the hydraulic conductivity, it is noted that the measurements made by slug tests in piezometers A and B, both being completely penetrating, show all the same scale parameter values. Hence for the correspondent *k* values the mean was considered, so as to obtain a single representative value for both *k* and scale parameters. The same was done for the slug tests performed in fully penetrating well number 11. The scale parameter values relative to the five considered tracer tests fall within a very limited range; therefore their mean value was assumed to be representative and the same thing was done for the corresponding five *k* values. By contrast, for the measurements carried out by pumping tests, the scale parameter values resulted were clearly different and included a very large range, so all the pairs of values (*k*, *R*) or (*k*, *V*) obtained by this method were considered. On the basis of these values the correspondent scaling laws *k* = *k*(*R*) and *k* = *k*(*V*) were determined and these are given by the following equations:

(11) $$\begin{array}{c} k=1\cdot 10^{-6}R^{0.245},\\ k=7\cdot 10^{-7}V^{0.122} \end{array}$$

while for both the relations the correspondent value of *R* ^2^ is equal to 0.833. The graph, with axes in logarithmic scale, of Figure 9 shows the trend of *k* versus *R* represented by the scaling law (11) and the corresponding 95% confidence intervals [65]; considering *V* as scale parameter it is possible to get a similar graph. The analogous scaling law of the hydraulic conductivity relative only to the values obtained by pumping tests, which are the majority, was also determined and this is described by the following equations:

(12) $$\begin{array}{c} k=1\cdot 10^{-6}R^{0.248},\\ k=7\cdot 10^{-7}V^{0.124}, \end{array}$$

both showing a coefficient of determination equal to 0.663. Equations (12) are very close to (11) and show trends practically coincident with that of the latter equations, but the value of the correspondent determination coefficient is clearly smaller. This trend is strongly influenced by the prevalence of the *k* and scale values obtained by pumping tests. However, the values of these parameters obtained by slug and tracer tests here considered improve substantially the fitting of the scaling law to the experimental values. It is also necessary to note that the parameter values obtained by slug tests here considered are exclusively relative to fully penetrating well number 11 and piezometers A and B, allowing, therefore, the exclusion of influences and errors due to methodological reasons [72].

Comparing relations (11) and (12) with (3), representing the model of Schulze-Makuch and Cherkauer [42], it is possible to notice that the values of the coefficient *c* obtained here are very close to those given by these authors for heterogeneous porous soils, while the values of the index *m* are slightly lower. Relatively to fractured geologic media, this comparison shows that the values of *m* relative to (8) and (9) are much lower, while those of coefficient *c* are generally higher [73].

These results seem to verify the existence, also for the considered aquifer, of a scaling behaviour of the hydraulic conductivity, confirming what was asserted by several authors [33–35, 38, 39, 74–79], some of whom verified a very good description of the scaling law by mathematical relations of the power type, independently of the specific method of *k* measurement utilized [42, 73].

The latter aspect has been carefully investigated by Schulze-Makuch and Cherkauer [42], who found that the scale dependence of hydraulic conductivity does not depend on the method of measurement. This is also verified in the present paper, as it is shown in the graphs of Figure 9, in which, excluding the single data relative to the tracer tests, both for those relating to slug tests and particularly for those relating to pumping tests, the scaling behavior is verified.

Commonly the observed scaling behaviour and hence the spatial variation of *k* are ascribed to the variation of heterogeneity of porous medium [32]; therefore the effective porosity plays an important role in this phenomenon.

The scaling behaviour can be extended to other parameters and also to the effective porosity. However, it should be noted that less is known about scaling of porosity (or storage coefficient for unconfined aquifers) and the studies on this subject are not numerous [37–39, 80, 81].

**Figure 10:**
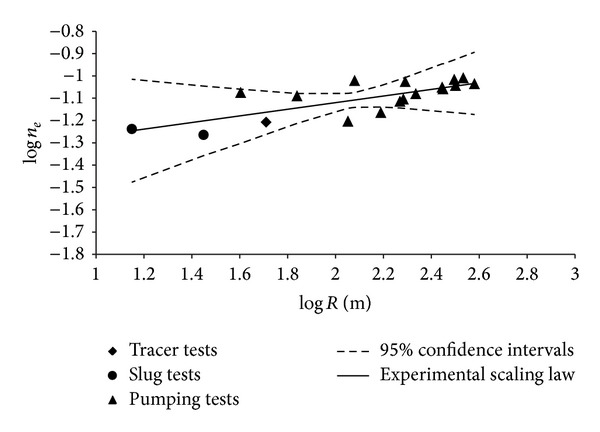
Trend of effective porosity versus scale for all field data sets.

In the light of what was above ascertained with (9), it is possible to retain that an increment of the hydraulic conductivity is strongly correlated with an analogous variation of effective porosity, which is with an increase of the interconnected pores. Therefore if the hydraulic conductivity shows a scaling behaviour, it seems reasonable to expect an analogous behaviour also from the effective porosity. This assertion needs a more careful experimental verification and further studies regarding the possible causes, because the scaling behavior of effective porosity is not always analogous to that of the hydraulic conductivity [37, 80]. With regard to the experimental aspect of the present paper, assuming as a representative scale parameter (*s*) both the radius of influence (*R*) and the aquifer volume (*V*) involved in the measurement, the *n*_*e*_ values show a certain scaling behaviour characterized by the equations

(13) $$\begin{array}{c} n_{e}=0.0383\cdot R^{0.1486},\\ n_{e}=0,0266\cdot V^{0,0743} \end{array}$$

which, analogously to (11), was obtained considering the mean values of *n*_*e*_ and *R* or *V* for the measurements carried out by slug tests and tracer tests, while for the aquifer tests all pairs of *n*_*e*_-*R* or *n*_*e*_-*V* values were considered, as above specified. The trend of *n*_*e*_ versus *R* described by (13), with corresponding 95% confidence intervals [65], is shown in the graph with axes in logarithmic scale of Figure 10; considering *V* as scale parameter it is possible to obtain a similar graph. The correspondent value of the determination coefficient is equal to 0.577, being therefore too low to be able to assert the existence of an effective scaling behaviour. Comparing the scaling law (13) with that obtained in a study of Fallico et al. [81], carried out on a laboratory model simulating an unconfined aquifer for which the scaling behavior of *n*_*e*_ was verified, it is possible to notice that the value of the coefficient *c* of (13) is less than that obtained in the study quoted, while for the index *m* one notes the opposite. The artificial aquifer of the laboratory model utilized in the study of Fallico et al. [81] constituted a sandy soil characterized by a value of *d*_10_ equal to 0.059, falling within the range of the samples examined here (see Table 2), but with a value of the coefficient *U* equal to 1.39, significantly lower than those shown in Table 2.

The scale effect of the porosity is scarcely investigated and not always analogous to that of the hydraulic conductivity. Furthermore, the variation of the porosity with the scale was not always found increasing, but in some cases it was revealed decreasing [37, 39, 80].

## 5. Conclusions

The effective porosity is a very important parameter to characterize the flow and transport phenomena in the porous media. The availability for an aquifer of a law such as *k* = *k*(*n*_*e*_) avoids the need to perform almost always expensive and time consuming measurement series. Nevertheless, considerable efforts and suitable measurement series are required to obtain an experimental law showing the trend of *k* versus *n*_*e*_ with validity to the scale required. The practical application of this law often requires the consideration of the field scale, able to represent both relatively small and large enough aquifer volumes and subject to the influence of heterogeneity average on these volumes. The problems of water flow and pollutant transport in porous media are generally related to portions of the aquifer at a scale greater than that of the laboratory and this makes most of the existing empirical and semiempirical formulas unsuitable to determine *k* from *n*_*e*_. Therefore, an experimental relation of this type between these two parameters must be determined on the basis of indirect field measurements of both hydraulic conductivity and effective porosity to be genuinely representative of an aquifer portion corresponding to the required scale.

For the confined aquifer of Montalto Uffugo test field, an experimental law, represented by (9), was obtained only by field measurements. This law can also be represented by (10), based on grain size analysis and on the formula of Vuković and Soro [41]. Several authors have already discussed relations of this type. However, most of these determine the law *k* = *k*(*n*_*e*_) on an experimental basis by hydraulic conductivity and effective porosity values determined in the laboratory on soil samples [3, 5, 6, 21]; other authors utilize experimental values obtained by field measurements for the hydraulic conductivity, but they utilize experimental values obtained in the laboratory on soil samples for the effective porosity [4, 22]. By contrast, the relationship determined in the present study exclusively by field measurements is able to provide more representative values of the parameters investigated for the aquifer volumes effectively involved in the measurements, even if its validity is limited to the soil type of aquifer examined. Excluding the carrying out of field measurements, with regard to the practical application of (10), the benefit is given by the possibility of using a relationship valid for field scales. The use of (9) and (10) can be considered particularly useful and preferable for sandy alluvial soils, with the same characteristics of that here considered (0.001 mm < *d*_10_ < 0.07 mm; *U* > 5;). In this regard, for the soil here examined the mean value of the grain uniformity coefficient (*U*) takes values near 22.29–32.03, as shown in Table 2. Nevertheless, one should consider that the grain size analysis for the examined samples showed that the grain size is variable in a rather large range. In fact, also if each soil sample shows a prevailing percentage of sand, the respective amounts of silt contained in them are always not negligible and sometimes important, with very low percentages of gravel varying to the different depths, while the content of clay becomes not negligible especially near the bottom. The characteristics of the porous medium where the examined aquifer is located are, however, those of the alluvial soils. Moreover, the experimental data utilized in the present paper allowed a scaling behaviour of the hydraulic conductivity that can be verified, considering all examined field data sets and also with only that relative to pumping tests, as shown, respectively, by (11) and (12) and the log-log graph of Figure 9. Regarding the existence of an analogous scaling behaviour for the effective porosity, also if conceptually possible, it did not have a strong experimental confirmation in the present paper, as (13) and Figure 10 show; therefore the necessity to carry out further experimental study on this matter is clearly apparent.

## Figures and Tables

**Table 1:** Identification number, depth from the ground level, and screen length of each well and piezometer relative to the confined aquifer of the test field.

	Wells						Piezometers	
Number	1	3	5	7	9	11	A	B
Well-top altitude (m a.s.l.)	154.76	154.78	154.74	154.68	154.77	154.53	155.00	154.85
Depth (m)	40	40	40	40	40	57	55	55
Screen length (m)	17	17	17	17	17	44	44	44

**Table 2:** Midpoint depth of the undisturbed soil samples examined in laboratory and relative meaningful results and parameters of the grain size analysis.

Drilling column piezometer A								Drilling column piezometer B							
Sample number	Depth (m)	Clay %	Silt %	Sand %	*d*_60_ (mm)	*d*_10_ (mm)	*U* = *d*_60_/*d*_10_	Sample number	Depth (m)	Clay %	Silt %	Sand %	*d*_60_ (mm)	*d*_10_ (mm)	*U* = *d*_60_/*d*_10_
1	14.15	7.2	23.3	63.5	0.27	0.003	93.10	1	11.90	3.5	16.5	77.8	0.4	0.025	16.00
2	16.65	9.6	28.9	60.5	0.17	0.002	70.83	2	12.40	2.8	12.2	77.8	0.7	0.023	30.43
3	17.85	16.7	45.7	37.6	0.05	0.001	51.00	3	13.30	3.2	16.8	80.0	0.4	0.015	26.67
4	19.05	5.2	23.4	68.0	0.30	0.005	65.22	4	14.50	4.9	17.1	78.0	0.42	0.0094	44.68
5	20.15	4.2	20.2	73.1	0.34	0.007	47.89	5	17.95	4.0	19.3	70.2	0.18	0.0025	72.00
6	21.45	5.4	22.5	67.7	0.20	0.007	29.41	6	22.95	5.8	11.2	80.0	0.63	0.059	10.68
7	23.20	5.8	21.2	72.2	0.18	0.006	32.14	7	34.65	2.3	9.3	80.6	0.68	0.084	8.10
8	27.15	3.5	16.1	80.0	0.22	0.020	11.00	8	38.75	2.5	6.0	85.0	0.24	0.026	9.23
9	29.85	23.6	50.8	25.4	0.02	0.001	24.00	9	41.00	4.5	8.0	80.0	0.53	0.048	11.04
10	34.75	3.7	17.4	71.3	0.40	0.012	33.33	10	42.75	4.2	7.6	86.7	0.53	0.071	7.46
11	40.65	6.5	27.1	65.4	0.24	0.004	61.54	11	44.15	1.0	8.8	89.4	0.44	0.073	6.03
12	43.05	1.7	9.3	88.5	0.28	0.053	5.28	12	48.00	2.9	6.9	90.2	0.43	0.06	7.17
13	44.25	1.3	8.6	88.1	0.40	0.060	6.67	13	50.45	3.0	8.0	89.0	0.41	0.08	5.13
14	47.45	0.9	15.9	83.0	0.18	0.040	4.50	14	52.65	2.8	6.2	91	0.23	0.004	57.50
15	48.45	1.6	12.6	84.1	0.37	0.030	12.33								
16	49.65	2.8	14.0	82.3	0.26	0.020	13.00								
17	50.95	3.5	14.6	81.0	0.25	0.025	10.00								
18	52.55	4.2	15.5	80.3	0.17	0.032	5.31								

Mean values	—	—	—	—	0.239	0.018	32.03	Mean values	—	—	—	—	0.44	0.041	22.29

**Table 3:** Meaningful statistical parameter values characterizing the considered data sets of hydraulic conductivity (*k*) and effective porosity (*n*_*e*_).

Parameters	*k* (m/s)			*n*_*e*_		
	Slug tests	Tracer tests	Pumping tests	Slug tests	Tracer tests	Pumping tests
*N*	15	5	15	15	5	15
Min.	1.63*E* − 06	1.83*E* − 06	3.28*E* − 06	5.44*E* − 02	4.50*E* − 02	6.25*E* − 02
Max.	3.27*E* − 06	6.00*E* − 06	5.78*E* − 06	5.78*E* − 02	8.26*E* − 02	9.77*E* − 02
Mean	2.56*E* − 06	3.54*E* − 06	4.64*E* − 06	5.71*E* − 02	6.20*E* − 02	8.50*E* − 02
Median	2.69*E* − 06	3.40*E* − 06	5.07*E* − 06	5.78*E* − 02	6.31*E* − 02	8.72*E* − 02
VAR	1.71*E* − 13	2.73*E* − 12	7.10*E* − 13	1.92*E* − 06	2.51*E* − 04	1.06*E* − 04
SD	4.14*E* − 07	1.65*E* − 06	8.42*E* − 07	1.39*E* − 03	1.58*E* − 02	1.03*E* − 02
SE	1.07*E* − 07	7.39*E* − 07	2.18*E* − 07	6.20*E* − 04	7.08*E* − 03	2.66*E* − 03
VC	1.62*E* − 01	4.67*E* − 01	1.82*E* − 01	2.43*E* − 02	2.54*E* − 01	1.21*E* − 01

**Table 4:** Values of the scale parameters *R* and *V* for each field measurement method considered.

Field measurement methods	*R* (m)	*V* (m^3^)
Slug tests		
Well no. 11	28.10	109093
Piezometers A and B	14.10	27468

Tracer tests (mean values)	51.28	363245

Aquifer tests	341.39	16102895
	112.60	1751775
	154.65	3304321
	40.17	222939
	278.74	10734478
	69.08	659395
	216.17	6456143
	380.53	20005994
	191.84	5084645
	313.25	13557392
	281.03	10911581
	195.45	5277809
	316.90	13874802
	185.90	4774645
	120.10	1992821
